# Treatment of Psoriasis by Rubber Sheeting

**Published:** 1877-03

**Authors:** 


Treatment of Psoriasis by Rubber Sheeting.—Dr. Dowse, of London, cures lepra and psoriasis by packing in rubber sheeting, without the use of any drug. This method of treating skin disease originated with Dr. Squire, who clad his patients in rubber undershirts and drawers. In a bad case of psoriasis Dr. Dowes first had the patient in a blanket bath for six hours. This was done by wrapping her in a blanket wrung out of hot water, and then covering with eight blankets, allowing her to drink freely of ice-water during the time. When she was removed all the scales had disappeared, leaving the body of a lobster redness. She was then enveloped in india-rubber sheeting, which was bandaged closely to the body. By this process the psoriasis might be said to have been converted into an eczema, for there was profuse discharge, with tendency to scabbing (for the first time). In a day or two the scales reappeared, but not to the same extent. On the 23d the bath was repeated, with similar good results. From this time the disease was manifestly checked, and, although there was the tendency to the re-formation of scales, it was in fact a pityriasis. On July 29th (for third time) I ordered her to be packed, and, by the 1st of August, there was scarcely a trace of the original malady, and at the hour of my writing (August 4th), the skin is perfectly healthy, and she says she never felt better in her life.—*Western Lancet.*

